# What Is a “Community Perception” of REDD+? A Systematic Review of How Perceptions of REDD+ Have Been Elicited and Reported in the Literature

**DOI:** 10.1371/journal.pone.0155636

**Published:** 2016-11-01

**Authors:** Stibniati S. Atmadja, Erin O. Sills

**Affiliations:** 1 Forests and Human Well-Being, Center for International Forestry Research, Addis Ababa, Ethiopia; 2 Department of Forestry and Environmental Resources, North Carolina State University, Raleigh, North Carolina, United States of America; Pacific Northwest National Laboratory, UNITED STATES

## Abstract

Reducing emissions from deforestation and forest degradation (REDD+) is expected to generate co-benefits and safeguard the interests of people who live in the forested regions where emissions are reduced. Participatory measurement, reporting and verification (PMRV) is one way to ensure that the interests of local people are represented in REDD+. In order to design and use PMRV systems to monitor co-benefits and safeguards, we need to obtain input on how local people perceive REDD+. In the literature, this is widely discussed as “community perceptions of REDD+.” We systematically reviewed this literature to understand how these perceptions have been assessed, focusing specifically on how individual perceptions have been sampled and aggregated into “community perceptions.” Using Google Scholar, we identified 19 publications that reported community perceptions of REDD+, including perceptions of its design, implementation, impacts, relationship with land tenure, and both interest and actual participation by local people. These perceptions were elicited through surveys of probability samples of the local population and interviews with purposively selected community representatives. Many authors did not provide sufficient information on their methods to interpret the reported community perceptions. For example, there was often insufficient detail on the selection of respondents or sampling methods. Authors also reported perceptions by unquantified magnitudes (e.g., “most people”, “the majority”) that were difficult to assess or compare across cases. Given this situation in the scholarly literature, we expect that there are even more severe problems in the voluminous gray literature on REDD+ not indexed by Google Scholar. We suggest that readers need to be cognizant of these issues and that publication outlets should establish guidelines for better reporting, requiring information on the reference population, sampling methods, and methods used to aggregate individual responses into “community perceptions.”

## Introduction

Reducing emissions from deforestation, forest degradation, and enhancement of forest carbon stocks (REDD+) is a global strategy to mitigate climate change. Much of the policy process and debate surrounding REDD+ has focused on its potential impacts on local people [1–3]. International guidelines for REDD+ implementation at the sub-national level include safeguards to ensure that the rights and aspirations of local communities affected by REDD+ initiatives are protected [4]. Various strategies, including participatory monitoring, reporting and verification (PMRV), have been proposed to more closely involve and benefit local people [5]. Many REDD+ initiatives follow standards that require varying degrees of community participation (e.g., [6]). However, the organizations implementing REDD+ have found it challenging to obtain full local participation, including identifying who truly represents the communities [7]. Better information on community perceptions of REDD+ is needed in order to develop strategies for local participation and monitor safeguards [8]. These perceptions cannot be directly observed or elicited in a survey, but rather require aggregating input from multiple informants. This in turn requires multiple judgement calls by researchers about how to aggregate the responses obtained from individuals and how to present the resulting summary measures of local perceptions of REDD+.

“Community perceptions” of REDD+ have been widely reported. These perceptions can inform both adaptive management of the REDD+ initiatives and the design of future REDD+ initiatives, including their institutional arrangements and methods for monitoring deforestation and its drivers. Community perceptions of the process, magnitude and cause of deforestation and forest degradation can also serve as the basis of PMRV or as a way to validate national MRV data. One source of data on community perceptions of REDD+ is research on early REDD+ initiatives, projects and demonstration activities (hereinafter referred to as “subnational REDD+ initiatives”) [9]. These initiatives offer the first glimpse of how REDD+ affects communities [10]. In many ways, REDD+ initiatives resemble previous conservation interventions, such as payments for environmental services (PES), community-based forest management (CBFM), integrated conservation and development projects (ICDP), and reforestation funded by the clean development mechanism (CDM) (e.g. [11–13]). However, REDD+ presents unique opportunities and challenges that may not be entirely captured in these past experiences [14]. In particular, REDD+ has a much stronger emphasis on performance-based approaches and on monitoring both carbon and social outcomes.

We systematically review the literature that reports “community perceptions” of subnational REDD+ initiatives. These reports are usually based on statements (perceptions, opinions or attitudes) elicited from people who live in and around those initiatives, aggregated or summarized in some way into community-level perceptions. As with all survey research, the exact wording of the questions and the interview format are likely to affect how individuals report their perceptions. However, in our review, we focus on the sampling and data collection methods, approach to analysis and aggregation of those data, and language used to report findings about “community perceptions.” Communities are comprised of multiple actors with multiple interests and perceptions [15, 16]. Research on community perceptions therefore involved fundamental choices about how to represent heterogeneous communities.

Our objective is assess whether published studies have provided adequate information on how the researcher drew conclusions about community perceptions from a set of individual responses. We address the following research questions:

1. *How have community perceptions of the implementation of subnational REDD+ initiatives been elicited*, *aggregated and reported?;* and
2. *Do published studies provide enough information on data collection and aggregation for the reader to interpret the reported community perceptions*?

## Eliciting and Aggregating Community Perceptions

The concept of “community perception” links individual experiences together into a collective experience. While perception refers specifically to “the process by which each individual selects, organizes, and evaluates sensory stimulations from the external environment to provide meaningful experiences for him or herself” ([17], p. 73), the literature on community perceptions often reports a mix of perceptions, opinions, and attitudes. The collective experience or beliefs of communities have been summarized as the fraction of the population that ascribes to particular opinions, the heterogeneity of perceptions that exist in the community, or reasons behind various perceptions. All of these require a sampling method to select the individuals to approach for information.

We broadly categorized sampling methods into two types:

1. *Purposive sampling*: Selects respondents (e.g. key informants) that fit into pre-determined categories based on the study’s purpose for either individual or group interviews (often called “focus groups”). Key informants are often selected for their deep knowledge or unique experiences, and ability to communicate their knowledge to the researcher [18]. These non-probabilistic sampling methods may be applied to extract cultural data, e.g. understanding processes rather than proportions [19]. Perceptions of key informants are not meant to represent community perceptions in a statistical sense [20].
2. *Probability sampling*: Respondents are chosen through some form of random sampling (e.g. random stratified or systematic) from a well-defined sampling frame. Each respondent in the sampling frame has a known, non-zero probability of being selected. Because the probability of being selected is known, data can be used to estimate population means, totals and proportions and the standard errors related to those estimates [21].

These two methods have different implications for research costs. With random sampling, there is always a chance that the randomly sampled respondents will not fall into the categories of interest, and thus larger samples of respondents are typically interviewed. Given a fixed budget, this requires greater efficiency, meaning that random sampling is often combined with structured questionnaires that facilitate data collection, entry and analysis. In practice, surveys using random samples tend to take more resources to implement compared to those using purposive samples, both because of the need to obtain a sampling frame and because of larger sample sizes [22, 23]. In comparison, key informant interviews are relatively low cost and quick to implement [24].

The methods also have different implications for the types of conclusions that can be drawn. Randomly sampled informants are typically interviewed independently, and thus their stated perceptions, opinions and attitudes reflect individual rather than social preferences, which may or may not be the most relevant for the question at hand [25]. On the other hand, a small group of key informants may be systematically different from the population they were meant to represent [26]. Although those key informants may be able to accurately describe structures and relationships in the larger population, it is often difficult for them to accurately describe the heterogeneity that exists in their community [27].

## Methods and Materials

### Definitions

In this study, the unit of observation was a ‘case’, or a publication that reported the methods and results of a study about local perceptions of REDD+. Specifically, we included publications that reported how REDD+ was perceived by “local” people, defined as people who lived in areas where subnational REDD+ initiatives or projects were being implemented. Their aggregated perceptions at the community level were often reported as “local community perceptions of REDD+.” We only included cases based on primary data from interviews and surveys of these local populations. This means that we screened out studies of REDD+ perceptions based solely on secondary data or solely on interviews with stakeholders who lived outside of the immediate areas where a subnational REDD+ initiative was implemented (e.g., project developers or NGO staff stationed in regional capitals, or national policy makers). When multiple stakeholders’ perceptions were reported in a case publication, we focused on the characteristics that pertained to how local perceptions were elicited and reported.

Because REDD+ is less than a decade old, many researchers have sought insights from forest conservation interventions similar to those proposed for REDD+, e.g. support for alternative livelihoods in Integrated Conservation and Development Projects (ICDP) or Payments for Environmental Services (PES) programs. We screened out publications that discuss lessons for REDD+ based on studies of “REDD-like” interventions. We focused exclusively on studies of REDD+ initiatives, which we defined as initiatives that (1) aimed to reduce emissions from deforestation and forest degradation and/or enhance carbon stocks (increase removals) primarily in existing forest in developing countries, and (2) planned to monitor and report or transact reductions in carbon emissions or increases in carbon stock in a quantified manner. Each case could report on multiple data collection methods, locations, and time periods.

### Systematic review methods

The cases examined in this paper were obtained through a literature search in Google Scholar (http://scholar.google.com/) using the Boolean search terms (“REDD+” and “perception”), yielding approximately 5400 results. Google Scholar indexed peer-reviewed literature, theses and dissertations, and grey literature. The literature search was conducted on 5–10 December 2013. This included in-press studies that were published in 2014. Search results were sorted by relevance according to Google Scholar’s algorithm. We went through this list consecutively. Based on the study abstracts or executive summaries, we retained studies that were written in English, addressed research questions related to community perceptions, and collected perception data from communities living in or affected by REDD+ subnational initiatives. We continued screening until no more literature was found in the next 150 search results after the last relevant case. A flow diagram of our literature search is available in S1 Fig. Using Google’s sorting algorithm reduced the influence of the researcher and the possibility of bias in selection of the studies. This search protocol yielded cases from the academic (e.g., scientific journal articles, dissertations) and gray (e.g., project reports) literature. It did not include non-scholarly materials (e.g., blogs, newspaper or magazine articles, pamphlets) or advocacy materials that may also contain statements on how communities perceived REDD+.

We systematically extracted the following types of information from each case:

1. *General study information*: Year published, publication type (e.g., journal article, grey literature), word count of full text (done by us), word limit imposed by publication outlet, study objectives/ research questions
2. *Context of community perception data*. Since perceptions change across space, time and elicitation techniques, we collected information on the way studies reported:
  - spatial information (e.g., country, locations, names and number of REDD+ initiatives and communities being studied)
  - temporal information (e.g., length of time spent in the field, timing of data collection relative to implementation of REDD+)
3. *Data collection methods*, including the way perceptions were elicited and the sampling methods. Specifically, we noted whether studies provided the following information.
  - *Site selection method*, for determining the REDD+ initiative and communities to study
  - *Reference population*, or the total population that the sample was meant to represent
  - *Respondent selection method*, through probabilistic or purposive approaches.
    We only documented methods used to interview local people, excluding information obtained from interviews of respondents outside the local communities, e.g. local government officials, or proponents of the REDD+ initiative who were not community members. For purposive sampling approaches, we recorded whether information on composition and number of respondents in focus group discussion (FGDs) or key informant interviews was reported. For probabilistic approaches, we recorded the number of observations and sampling rate. We assessed the adequacy of sample size to represent the reference population based on the minimum sampling size based on Eq 1 (adapted from [28]):
    $$n=\frac{Np\left(1-p\right)}{\left(N-1\right)\frac{c^{2}}{z^{2}}+p\left(1-p\right)}$$ (1)
    where N = Size of the reference population (provided by the author); p = estimate of the proportion of individuals with characteristic of main interest in the survey, set at 0.5 to reflect the highest level of heterogeneity c = target margin of error set at 0.10 (more generous than the 0.05 level suggested by many survey sampling tools e.g. [29]), and; z = z-score. We used a benchmark z-score (z) of 1.96, following the scientific convention of setting the confidence level at 95% ([28], p.57).
  - Caveats about data collection that may have affected the results, such as difficulties in language, establishing rapport, and access to both male and female respondents
4. *Evidence base for community perceptions of REDD+*. In the ‘results’ section of each case, we documented the source of evidence used to support reported results on community perceptions of REDD+, such as proportions of respondents, key informant statements and number of FGDs.

The above parameters were extracted mainly from the “methods” and “results” sections in each case, using MaxQDA v.10 software [30]. Coded segments were analyzed and categorized. If the methods and results sections were not clearly marked, analysis was applied to statements of the case’s data collection procedure and its findings (See S1 File for the full dataset). We relied on the vote-counting method [31] to summarize findings. We adopted the following terms to denote units of analyses: reference population (the general population considered by author to be part of/affected by the REDD+ initiative), study communities (community groups or villages in the reference population from which data were collected), study households and individual respondents.

We used word counts as a proxy for limitations on length that were imposed by some publication outlets, which could have discouraged full reporting on data collection, aggregation and analysis methods. Two metrics were used: (i) Word count of the full text, including abstract, tables and references (See S1 File for details), and (ii) Word limits imposed by publication outlets as published in their websites. We note that Google Scholar included publications of all lengths, including theses, working papers, conference papers, as well as peer-reviewed journal articles.

## Results

### General study information

Nineteen cases passed our screening criteria. These were identified within the first 180 search results (i.e., we found no additional cases in search results 181 to 331) [8, 32–49]. These cases illustrated the wide range of ways that sampling procedures were used and communicated in studies on community perceptions of REDD+. We found three types of cases: (i) purely qualitative analytical methods based on purposive sampling; (ii) purely quantitative analytical methods based on probabilistic sampling; and (iii) mix of qualitative and quantitative analytical methods, using data collected from purposive and probabilistic samples. Most cases were peer-reviewed journal articles (7 cases) or master’s theses (5 cases). The remainders were reports (2 cases), working papers (3 cases), conference papers (1 case), and book chapters (1 case). Except for one undated publication, they were published from 2009 to 2014. The largest number of cases (6) was published in 2013. Four cases were published in 2014 as of March 2014.

Shorter publications (with lower word counts) may be less likely to report the information we were seeking because of word limits. The median word count was 14,500. Three studies were particularly short with less than 7,000 words [8, 38, 40]. All cases that were the journal articles had word limits ranging from 4,500 to 10,000 words.

The stated objectives and research questions motivating these cases could be grouped into five main themes: (i) Expected or actual effect of REDD+ on communities (benefits, costs, changes) (8 cases); (ii) Tenure issues (8 cases); (iii) Design and implementation of initiatives (8 cases); (iv) Interest in or feasibility of REDD+ (6 cases), and (v) Quality of community participation in REDD+ (5 cases). The question of how REDD+ impacts local people was an important part of this literature. In six of the eight cases that aimed to study REDD+ impacts, this question was asked prior to any REDD+ payments being made. In some cases (e.g. [42]) local communities still had very little awareness that a REDD+ initiative was being implemented in their area. In such cases, authors focused on the proponents’ plans to deliver benefits (e.g., [41]), eliciting local people’s hopes or worries (e.g., [8]), devising hypothetical scenarios based on historical data (e.g., [35, 42, 46]), or constructing conceptual models linking baseline conditions to final outcomes (e.g., [45]). In the remaining 2 cases, the initiative were far along enough for impacts to be perceived (i.e., [43, 49]). Studies on tenure characterized local people’s perceptions of tenure conditions and the factors that affect tenure. In this context, REDD+ was analyzed as a factor that affected tenure security (e.g., [39, 48]) or was affected by it (e.g., [45]). Some of these studies also elicited information from proponents, to understand how they planned to address tenure challenges (e.g., [34, 47]). The topics that could be studied depended on how far REDD+ implementation has gone. In most cases (17 of 19 cases), the actual outcomes were still unknown because REDD+ initiatives were in early stages of implementation and not yet offering REDD+ payments. Under these conditions, respondents could express their views on how initiatives were governed and organized (e.g., [41, 44]), communicated with local people (e.g., [34]), planned to improve livelihoods (e.g.,[38]); share benefits (e.g., [36]) and accommodated gender issues [36]. Some cases focused on participation of specific social groups, such as women [36] and indigenous communities [35, 39, 42, 48]. Low awareness resulted in low participation [40], and in some cases, awareness of the REDD+ initiative was so low that it was difficult to elicit respondents’ willingness to participate [42]. In the 2 cases where REDD+ payments had been made (i.e., [33, 46]), the payments were distributed in the form of community-based projects.

### Context of community perception data

#### Spatial information

Countries studied most often were Tanzania (7 cases), Indonesia (5 cases) and Brazil (5 cases). Eleven cases focused in 1 REDD initiative, 5 cases consist of single-country studies with multiple initiatives (up to 5 REDD+ initiatives), and 3 cases were multi-country studies with 4–6 countries and 9–22 REDD+ initiatives. Of the 8 cases on multiple initiatives, 7 (including all of the multi-country cases) were part of the REDD+ global comparative study conducted by the Center for International Forestry Research. The spatial scale ranged from an in-depth ethnographic study of one village (i.e., [32]), to multi-country studies involving 71 villages across 6 countries and 3 continents (i.e., [39]). All cases clearly identified both the names and the countries of the initiatives being studied, except for one that covered 22 initiatives (i.e. [39]).

#### Temporal information

The length of time spent in the field may be correlated with data reliability, because more time in the field likely means more time to triangulate information and to build trust that helps reveal ‘true’ perceptions. That is, all else equal, a researcher who spends more time in a village is more likely to understand the underlying meaning of statements made by local people on how they perceive REDD+ implementation. Among the cases we analyzed, we estimated the length of time spent conducting data collection ranged from 3 weeks to 3 years. It is difficult to estimate the amount of time spent per community, since it is possible that a study has several field teams collecting data in several places simultaneously. Some cases did not provide information on the length of time spent in data collection (4 cases) and/or the number of study communities (2 cases).

Another aspect is the timing of data collection relative to the phase of the REDD+ initiative. Perceptions of local people may differ significantly depending on whether they have heard about the initiative, participated in activities, or received direct benefits. Hence, it is important to understand where data collection fits in the timeline of the REDD+ initiative. All cases except for one provided the month and year of data collection, implementation milestones, or both. Implementation was typically described as either ‘early implementation’ (e.g., defining the boundary of the REDD+ intervention area, communicating about REDD+ to local people) or in reference to actual or planned delivery of performance-based payments.

Information on these temporal factors could be difficult to find. Three cases mentioned the year of data collection as a footnote or only in the abstract. Some cases referred euphemistically to the early stage of REDD+ implementation, e.g. “initial field experiences” [38], “baseline research” [37] or “early process outcomes” [39, 47]. Others provided the temporal context in a separate section describing implementation, letting readers independently piece together the information. For example, Bradley et al. [36] collected data in April 2012, while noting in a separate section that the REDD+ initiative “goes to market in 2012”.

### Sampling methods

This section describes the sampling methods used in our cases. Within each REDD+ initiative, data collection was stratified by settlements/villages (13 cases), community forest user groups (3 cases) or household (1 case). The remaining two cases (i.e., [33, 41]) reported results from community members but did not provide information about the community groups from which they were elicited. At the household or individual levels, there were two main approaches to selecting respondents: probabilistic or purposive. Most (11 cases) used both approaches for different elicitation methods; two cases only used random selection; and six only used purposive selection. In the 19 cases, we observed that random selection was only used with structured or semi-structured interviews of individuals (e.g., interviews with household heads), while purposive selection was used both for those interview methods and for unstructured dialogues and semi-structured interviews with groups (e.g. focus group discussions).

#### Site selection

Criteria for selecting REDD+ initiatives, communities and individual respondents included logistical/practical reasons (e.g., distance, willingness to participate in study, level of REDD+ implementation) and substantive reasons (e.g., ecosystem types, ethnicity, and REDD+ approach). At the level of the REDD+ initiative, only one case described a process of selection from a larger population of REDD+ projects [33]. Level of advancement in REDD+ was the most common criteria for choosing initiatives (16 cases), followed by criteria related to socioeconomic characteristics (4 cases), REDD+ approach (4 cases) or ecological/forest characteristics (2 cases). For selection of study communities, exposure to REDD+ implementation was the most common criteria (8 cases). Two cases [46, 49] followed a sampling procedure designed to capture variation in the level of involvement across study communities, while the other six cases only specified that they focused on communities involved in REDD+. Nine cases provided no information on how they selected community groups to study.

#### Study or reference population

Regardless of the sampling method, we tried to find information on the reference population from which respondents were sampled, at each level of stratification. At the community level, 5 cases specified both the number of sampled communities and the total number of communities in the REDD+ initiative. Three other cases chose a sampling frame of 15–16 communities within the REDD+ initiative, and then sampled among them. The remaining 11 cases provide no or partial information on the total population, sampling frame, or sample size. In these cases, the question of “what does your sample represent?” cannot be answered adequately. In some cases, (e.g., [8, 47]) authors state what their studies did not represent.

#### Respondent selection method

In 13 cases, respondents or households were selected randomly. Among the cases that used probabilistic sampling, the minimum required sample size could be determined only if both the number of respondents and the size of the reference population (or the sampling frame) were specified. In 8 of the 13 cases, the size of the sampling frame (e.g., total number of households in study villages) was not stated. Of the remaining 5 cases, four (i.e., [37, 38, 40, 43]) met the minimum sample size in Eq 1.

Most of the cases (16 of 19 cases) used purposive sampling methods, namely FGDs (4 cases), key informant interviews/dialogues (1 case) or both (11 cases). Of the 16 cases using purposive sampling methods, 6 provided complete information about the composition (e.g., gender, age, position) and number of their respondents. Among 12 cases that used key informant interviews, 5 provided complete information (composition and number), 6 gave only the composition of respondents, and 1 gave only the number of respondents. The 15 cases that used FGDs are divided into those that provided information on the composition and number of participants (8 cases), composition only (2), number only (1) or neither (4).

#### Caveats about data collection

Eleven cases presented caveats about their data collection and sampling processes. Common caveats included language barriers (4 cases), factors that could bias data collection or sampling (7 cases), and issues with field assistants (4 cases). It is likely that similar caveats apply to the remaining 8 cases as well, but were not mentioned. These caveats were often found in sections on ethical reflections and study limitations, which are part of the methodological narrative in ethnographic studies and masters theses (e.g. [32, 35, 42]).

### Evidence base for community perceptions of REDD+

Ideally, studies drew conclusions about community perceptions based on evidence from the data they collected from individuals. Many cases based their conclusions on aggregated data such as the proportion of respondents (9 cases) or conclusions/majority opinions in focus group discussions (8 cases). Individual statements were also used to represent community perceptions. These included statements from key informants (4 cases), individual statements during focus group discussions (6 cases), and insights from unstructured dialogues (3 cases). Furthermore, statements about a community’s perception of an issue were often made based on data from unknown sources, the most common being statements made by ‘community members’ without reference to the data collection method. It was also common for cases to evoke magnitude (e.g., “most people”, “the majority”) without qualifying what that means (12 cases).

## Discussion

The sampling method is a critical component of research on community perceptions because communities are *not* homogenous. They consist of individuals with different views, which must be aggregated in some form to represent ‘community perceptions’. Many cases (9 of 19) did not provide information about how they chose study communities. Of the remaining 10 cases, 3 used focus group discussions but did not provide information on the composition of participants. Hence, for most cases we could know gauge whether the researcher captured heterogeneity within their reference population. Perceptions are highly contextual. In the context of REDD+ and PMRV, they depend on factors such as the progress of the REDD+ initiative, environmental and social conditions, and the researcher’s elicitation methods. Thus, readers need information on this context, including “when, where, and how” a study was conducted. Among the 19 cases reviewed, there are many omissions of this basic information. At minimum, in a study of community perceptions, it should be standard practice to describe the community, at least in general terms that do not compromise respondent confidentiality. Yet we found two cases lacking any description of the communities whose perceptions were being described. The timing of data collection relative to REDD+ implementation milestones should be transparent, because perceptions can change as people update their views based REDD+ activities.

Various methodological choices can influence findings about what communities think and feel about REDD+. For example, site selection criteria may be correlated with perceptions of REDD+. In some of the cases we reviewed, this was taken into account in the sampling strategy, i.e. by choosing villages with variable levels of involvement in a REDD+ initiative [49]. Seven cases explained how their data collection methods could influence their results, which suggests a degree of transparency about this issue in the literature. However, 12 of the 19 failed to provide information on site selection at either the initiative or community level. This constitutes a large proportion of our dataset, suggesting a worrying tendency to overlook the importance of such contextual information.

Cases with shorter word counts were most likely to omit important elements of the data collection methods that we evaluated in this paper. For example, of the 9 cases that did not provide information on community selection criteria, 5 were journal articles (with word limits), and another 2 had less than 10,000 words. Of the 8 cases that did not include any caveats about their data, 4 were journal articles and another 2 had less than 7,000 words. While word limits may partly explain the gaps in descriptions of the methods, some of the missing information could be presented very concisely. For example, the number and composition (e.g., age, gender) of FGD participants (missing in 7 cases), sizes of the sample and reference population (missing in 8 cases), and the length of time researchers were in the field (missing in 4 cases).

In disciplines such as medicine, similar oversights would be unthinkable. Reports on clinical trials always specify the number of participants in the trial. In another dimension of REDD+, studies of forest carbon always provide information on the species composition of trees in their sampling area. Thus, there appears to be a particular lack of attention to these details among authors and/or publication outlets concerned with community perceptions.

We only have evidence on numbers and trends, but can only speculate on their causes. It could be that within this field, honesty is not the best policy. Providing methodological information that can reveal the weaknesses of the study’s data collection in representing the community as a whole could lead to paper rejections. Yet we wonder why *not* providing this information is still acceptable, including among peer-reviewed journal articles. But since we did not review rejected papers, we cannot conclude either way. Another possibility is that unlike clinical trials or forest mensuration, which rely on probabilistic sampling, studies on human perceptions is dominated by the use of purposive sampling. Yet regardless of scientific discipline, studies need to provide information on the origins of their data (temporally and spatially) and potential causes of bias. Our paper used different sets of criteria for purposive and random sampling to level the playing field, and found basic information on data collection are still missing. Our own study is also with its limitations. We relied on one database (Google Scholar), whose search algorithm and database coverage is unclear, which may lead to biases in our findings due to inadvertent omission of certain publication types. We collected data until December 2013; since then, there may be additional publications on community perceptions of REDD+ that could complement our dataset. We are still confident that our general conclusions would remain valid, because there has not been substantial shifts in the way data collection methods are reported in the REDD+ literature since then.

Carefully designed and transparent sampling methods can help ensure that conclusions about local people’s perceptions are not misunderstood, misrepresented and taken out of context. Most cases on community perceptions of REDD+ projects that were analyzed failed to provide enough information to answer the question: “Who did you sample? What does your sample represent?” Basic information such as the size of the population being sampled and clear descriptions of key informant characteristics were missing from the majority of cases being studied. Without this information, readers cannot ascertain the extent to which results can be generalized. Nevertheless, many of these cases occasionally use generalizations that apply to populations larger than the respondents they interviewed. This is expressed by misusing aggregated units of observations like “households”, “communities”, or “women” in reporting results, which imply results are representative of the entire unit. This oversight does not seem to be purposefully done to conceal information or overreach one’s ability to generalize. It may exist because of language problems (e.g., English as a second language), the need for brevity, or a lack of attention to this aspect in the publication process.

Ethnographic studies and master’s theses provide excellent examples of the necessity of caveats and personal reflections in helping readers understand the limitations of each study. Those studies mentioned gender biases due to cultural barriers between researcher and respondents, language barriers, logistical constraints, and personal beliefs that may influence the way researchers interpret field information. They should always be noted because they could be a source of bias during data collection and analysis. It is highly likely that such constraints are also found in other publication formats that are less likely to include these caveats, possible due to page constraints and convention.

More speculatively, missing sampling information could be an attempt by authors to minimize criticism of their data quality. In such cases, the research community needs to come to terms with the conflicting demands of transparency and rigorous data collection standards. In reality, data collection of community perception is often determined by real-world considerations such as ease of access to communities, research budget, researchers’ field experience, and political agendas of the researcher or research partners (e.g., local NGO guides). In the name of transparency, would it be better to encourage researchers to publish these considerations and accept the consequences in data quality?

There are, however, some minimum standards of transparency that we feel should be encouraged for studies on community perceptions. Studies should clearly state what their sample does and does not represent. This includes: the reference population where the study’s results can be applied, description of sampling methods and respondents, and caveats/limitations. Perception studies that rely on key informants should describe their informants in terms of their role in the community, interests in REDD+ activities, and their relationship to the REDD+ initiative, and the number of people who were interviewed. This enables readers have a way to judge, or at least get a taste of what kind of group those informants are coming from, and what they represent in the community. Authors should be cognizant that representativeness can be unduly implied through their choice of words. This can be overcome by using words such as “study villages”, or “respondents” that restrict results to the reference populations, and framing the results by the method of data collection (e.g., “the focus group discussions revealed that…”).

We recommend that researchers be honestly imperfect by providing enough information for readers to fully judge the representativeness of their data and conclusions. In response, publication outlets should encourage authors to be transparent about their data’s limitations, and provide best practice guidelines for responsible reporting on human perceptions in general. This includes providing information on the reference population, sampling methods, and statistics used to aggregate individual responses into a ‘community perception’.

Our most important message concerns the publications that have *not* been reviewed in this paper. Information about community perception on REDD+ can be found in more popular forms of publications (e.g., websites, newsletters, magazine articles, advocacy material) that are rarely expected to provide information on data collection and sampling methods. Some of these contain strong messages about REDD+ on behalf of forest-dependent communities (e.g. [50, 51]). As the REDD+ process continues to unfold at the global level and the interest in community perceptions of its implementation grows, this study provides a cautionary note. Readers of the REDD+ literature of *any kind* must be critical about how much they can generalize from what they read, and authors should provide enough sample and methodological information to allow readers to judge.

## Supporting Information

S1 Fig Flow diagram of literature search. (DOC)

S1 File Summary of data collection and sampling methods of studies on community perceptions of REDD+. (XLSX)

S1 Table PRISMA 2009 Checklist. (DOC)
