# Correction: What Is a “Community Perception” of REDD+? A Systematic Review of How Perceptions of REDD+ Have Been Elicited and Reported in the Literature

**DOI:** 10.1371/journal.pone.0173547

**Published:** 2017-03-02

**Authors:** Stibniati S. Atmadja, Erin O. Sills

There are errors in the Funding section. The correct funding information is as follows: This research is part of the CGIAR Research Program on Forests, Trees and Agroforestry and the Global Comparative Study on REDD+, with funding support from the USAID (grant number MTO069018, http://www.usaid.gov/), the Norwegian Agency for Development Cooperation (Norad) (grant number QZA 12/0882 and QZA-16/0110 No. 1500551, http://www.norad.no/), the International Climate Initiative (IKI) of the German Federal Ministry for the Environment, Nature Conservation, Building and Nuclear Safety (BMUB) (grant number KI II 7–42206-6/75, https://www.international-climate-initiative.com), and the CGIAR. The above mentioned funders had no role in the study design, data collection and analysis, decision to publish, or preparation of the manuscript.
