# Supplementary material for: What Is a “Community Perception” of REDD+? A Systematic Review of How Perceptions of REDD+ Have Been Elicited and Reported in the Literature
Source: PLoS One. 2016 Nov 1;11(11):e0155636. doi: 10.1371/journal.pone.0155636 (PMC5089688; doi:10.1371/journal.pone.0155636)
Supplement: S1 Fig — (DOC) [file pone.0155636.s001.doc]

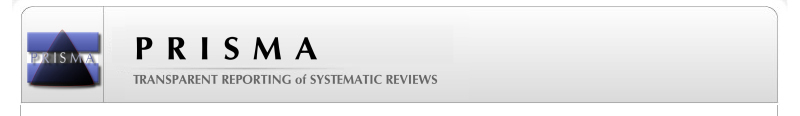
**PRISMA 2009 Flow Diagram**

**Screening**

**Included**

**Eligibility**

**Identification**

Records identified through database searching
(n = 5400)

Additional records identified through other sources
(n = 0 )

Records after duplicates removed
(n = 5400 )

Records screened
(n = 330 )

Records excluded
(n = 5170 )

Full-text articles assessed for eligibility
(n = 180 )

Full-text articles excluded, with reasons
(n = 161 )

Studies included in qualitative synthesis
(n = 19 )

Studies included in quantitative synthesis (meta-analysis)
(n = 19 )
